# A 3D stereotactic atlas of the adult human skull base

**DOI:** 10.1186/s40708-018-0082-1

**Published:** 2018-05-31

**Authors:** Wieslaw L. Nowinski, Thant S. L. Thaung

**Affiliations:** 1 John Paull II Center for Virtual Anatomy and Surgical Simulation, Cardinal Stefan Wyszynski University, Woycickiego 1/3, Block 12, Room 1220, 01-938 Warsaw, Poland; 2 Department of Radiology, University of Washington, Seattle, WA USA; 3 Mae Tao Clinic, P.O. Box 67, Mae Sot, Tak 63110 Thailand

**Keywords:** Skull base, Electronic atlas, Digital models, Skull, Brain, Stereotactic atlas

## Abstract

**Background:**

The skull base region is anatomically complex and poses surgical challenges. Although many textbooks describe this region illustrated well with drawings, scans and photographs, a complete, 3D, electronic, interactive, realistic, fully segmented and labeled, and stereotactic atlas of the skull base has not yet been built. Our goal is to create a 3D electronic atlas of the adult human skull base along with interactive tools for structure manipulation, exploration, and quantification.

**Methods:**

Multiple in vivo 3/7 T MRI and high-resolution CT scans of the same normal, male head specimen have been acquired. From the scans, by employing dedicated tools and modeling techniques, 3D digital virtual models of the skull, brain, cranial nerves, intra- and extracranial vasculature have earlier been constructed. Integrating these models and developing a browser with dedicated interaction, the skull base atlas has been built.

**Results:**

This is the first, to our best knowledge, truly 3D atlas of the adult human skull base that has been created, which includes a fully parcellated and labeled brain, skull, cranial nerves, and intra- and extracranial vasculature.

**Conclusion:**

This atlas is a useful aid in understanding and teaching spatial relationships of the skull base anatomy, a helpful tool to generate teaching materials, and a component of any skull base surgical simulator.

## Introduction

The base of skull, or skull base, forms the floor of the cranial cavity and separates the brain from other structures of the face and neck. It is composed of five bones: ethmoid, sphenoid, occipital, frontal, and paired temporal. The skull base is subdivided into three distinct regions called fossae: the anterior, middle, and posterior cranial fossae. The base of skull not only separates the intracranial content and the facial compartment from the upper respiratory and digestive tracts, but it also enables the passage of vital neurovascular structures entering and exiting the brain. The skull base region is anatomically complex and poses surgical challenges for neurosurgeons and otolaryngologists because of proximity to important structures, such as the brain, cranial nerves, orbits, and carotid arteries, among others. Moreover, the skull base provides access to hardly accessible intracranial lesions located adjacent to the brainstem [1]. Therefore, knowledge of the normal and variant anatomy of the skull base is essential for effective surgical treatment of disease in this region.

The skull base has been comprehensively described in many textbooks [2–4], Gray’s anatomy [5–7], and print atlases [1, 8, 9]. This kind of material is illustrated with drawings, tomographic scans and pictures of cadaveric specimens where the images are typically unlabeled. The skull base region is also available in some electronic atlases of the head and neck [10–14]. These applications vary from medical illustrations to animations to limited three-dimensional (3D) models. Another approach enabling to study the skull base region is to generate 3D printed models [15]. Moreover, some applications employ virtual reality systems for training and planning skull base surgery [15, 16].

Although many textbooks describe the skull base region well illustrated with drawings, scans, and photographs of anatomical dissections as well as animations and other electronic media have been developed for studying the skull base region, a complete, truly 3D, electronic, interactive, realistic, fully segmented and labeled, decomposable, and stereotactic atlas of the skull base has not been created yet. Therefore, there is a great need for a 3D atlas covering the practical neuroanatomy of the skull base region. The goal of this work is to create a 3D electronic atlas of the adult human skull base along with interactive tools for structure manipulation, exploration navigation, and quantification.

## Materials and methods

A 3D atlas of the adult human skull base region has been created from 3D digital virtual models of the skull, brain, cranial nerves, intracranial vasculature, and extracranial vasculature. These models have been built earlier from in vivo multiple 3 and 7 Tesla (T) magnetic resonance imaging (MRI) and high-resolution computed tomography (CT) scans of the same normal, male head specimen. The models have been incorporated into the 3D stereotactic atlases of the skull [17], brain [18], cranial nerves [19], intracranial vasculature [20], and extracranial vasculature [21].

A process of anatomical model building was, generally, similar for each model, although varying in data acquisition, modeling methods, building suitable tools, and validation. This process included the following steps: in vivo scan acquisition, scan segmentation, scan parcellation into individual structures, 3D surface modeling, 3D model simplification (decimation or compressing), 3D surface editing, 3D model color-coding, 3D object naming based on *Terminologia Anatomica* [22], and model validation. The anatomical models were placed in the Talairach stereotactic coordinate system [23], and a readout of distances and the stereotactic coordinates (posterior–anterior, inferior–superior, and right–left) were provided.

In order to build a 3D model of the skull, a high-resolution spiral CT scan was acquired with 526 axial slices of 0.75 mm thickness, 0.5 mm increment, 512 × 512 matrix and 0.46875 × 0.46875 mm^2^ pixel size. The skull was originally parcellated into 29 individual bones, reconstructed in 3D, and validated as featured in [17]. The skull was also cut anterio-posteriorly with its superior part removable in order to enable exposure of the skull base region. The 3D skull base model comprises 5 bones: ethmoid, sphenoid, occipital, frontal, and paired temporal, as well as foramina and canals of the skull base and other bony structures along with landmarks typical to this region. The size and shape of the foramina and canals were carefully processed to fit the blood vessels and cranial nerves that pass in and out of them.

The brain model was constructed from a 3 T magnetization-prepared rapid gradient echo (MP-RAGE) scan with a 224 × 300 × 320 resolution and a 0.8 × 0.8 × 0.8 mm^3^ voxel size. The brain was segmented by means of a dedicated contour editor, a 3D model constructed, and the cortex parcellated into lobes, gyri, and sulci [18].

In order to build a 3D model of the intracranial vasculature, multiple 3 and 7 T magnetic resonance angiography (MRA) scans were acquired, including 3D time of flight (the main sequence with a 384 × 320 × 272 resolution and a 0.52 × 0.52 × 0.5 mm^3^ voxel size), spoiled gradient recovery, and susceptibility weighted imaging. The vessels were extracted using a dedicated vascular editor [24] and modeled as tubes as explained in [25]. The vascular editor provides functions for a rapid extraction of vessels on the orthogonal axial, coronal, and sagittal planes and constructing centerline and 3D polygonal tubular surface models.

The extracranial vasculature was created in a similar way from the same MRA scans as the intracranial vasculature and additionally from a 3D phase contrast MRA neck scan with a 352 × 352 × 261 matrix and a 0.568182 × 0.568182 × 1.0 mm^3^ voxel size [21].

The cranial nerves (CN) extracted initially by means of the vascular editor from the MP-RAGE scan were also modeled as tubes, and further extended and fine-tuned as described in [19]. The CN model contains all 12 pairs of CN I-XII (with over 600 segments) as listed in [19].

In order to browse, explore, navigate, quantify, and manipulate the skull base models, the atlas is equipped with a user-friendly browser with dedicated interaction. Browsing and exploration is facilitated by: (1) the assembling (compositing) and disassembling (decompositing) mechanism with an individual and group selection allowing the investigator to build any region of interest or (sub)system and to present a local anatomy within its global context; (2) virtual brain dissections in seven directions to see through the brain; (3) real-time structure and scan manipulation; (4) 3D labeling of surface and sectional MRI anatomy; (5) structure highlighting, (6) interaction combined with animation; and (7) quantification with a readout of distances, vessel diameters, and the stereotactic coordinates.

## Results

**Fig. 1:**
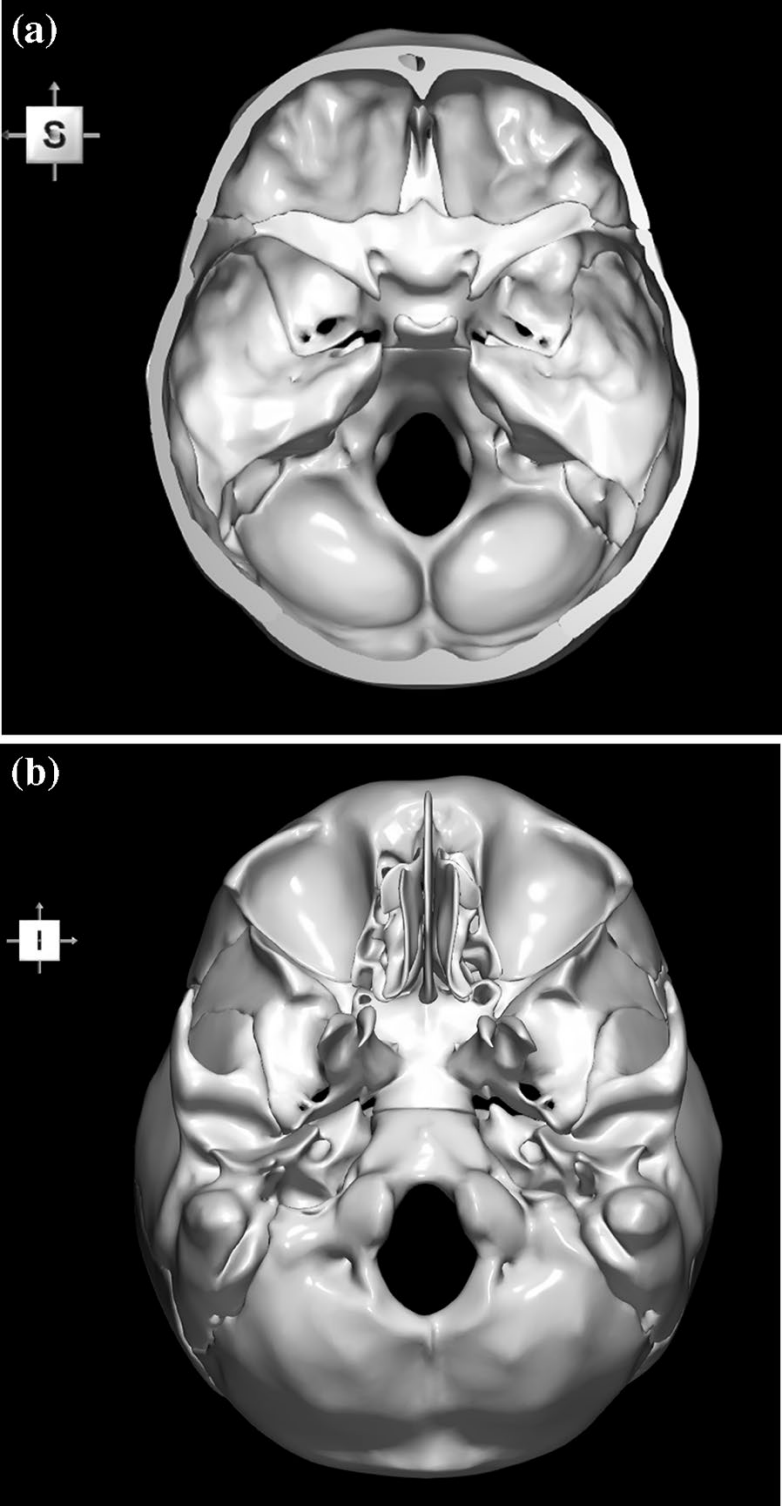
Skull base: **a** internal surface (superior view); **b** external (basal) surface (inferior view). The orientation box located in the top-left corner indicates the viewing direction (*S* superior, *I* inferior, *A* anterior, *P* posterior, *L* left, and *R* right)

**Fig. 2:**
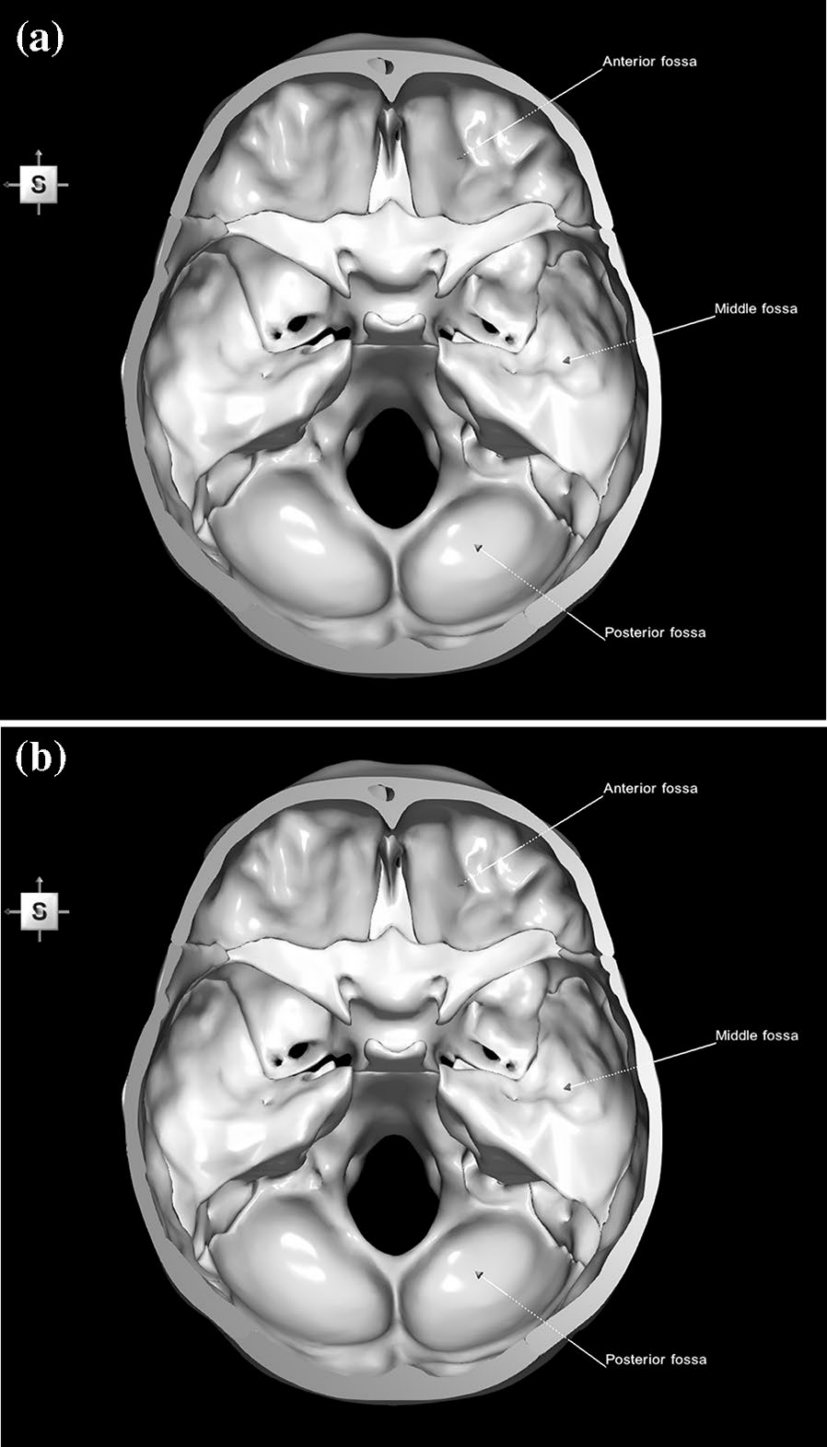
Internal surface of the skull base labeled with: **a** anterior, middle and posterior fossa; **b** color-coded five bones

**Fig. 3:**
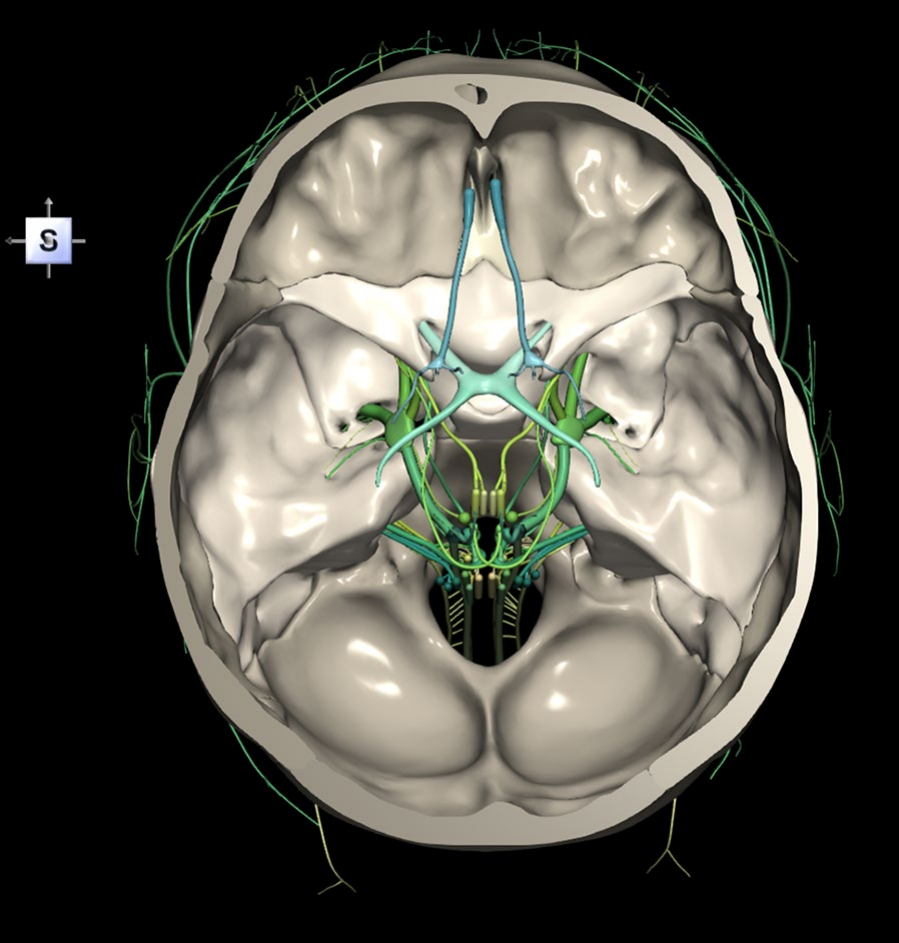
Skull base along with foramina, canals and the cranial nerves CN I–XII

**Fig. 4:**
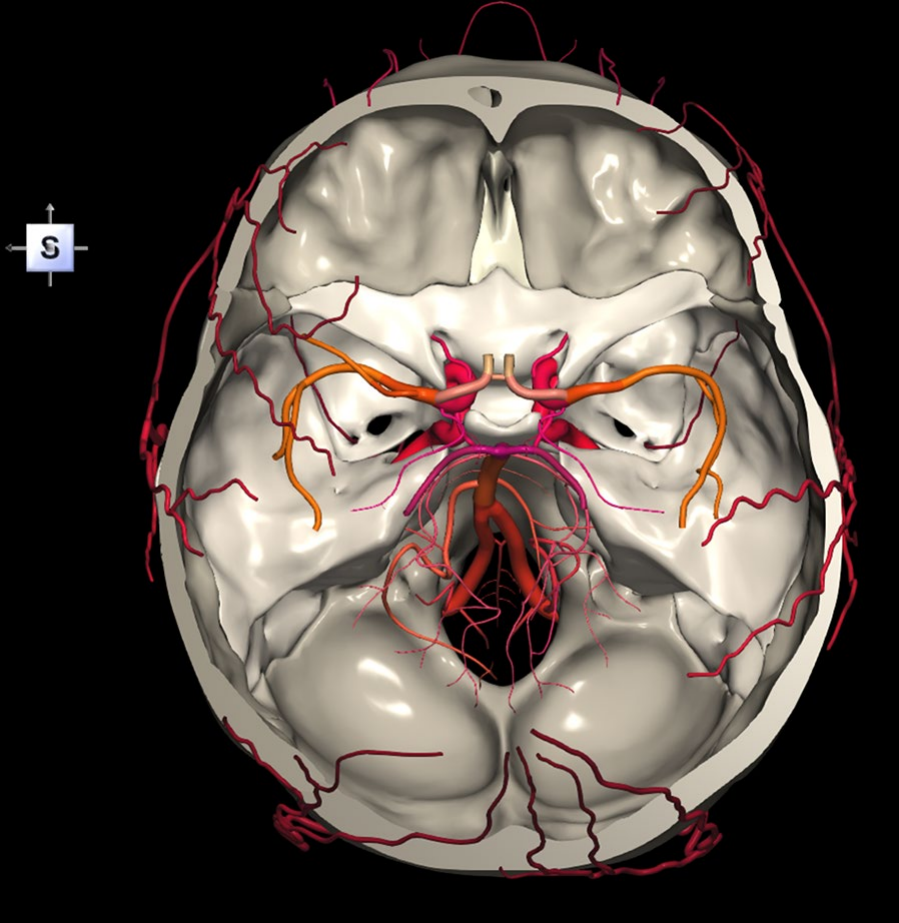
Skull base along with selected intracranial arteries (internal carotid artery, vertebral artery, basilar artery, middle cerebral artery (M1 and M2 segments), posterior cerebral artery (P1 and P2 segments) and circle of Willis) and selected extracranial arteries

**Fig. 5:**
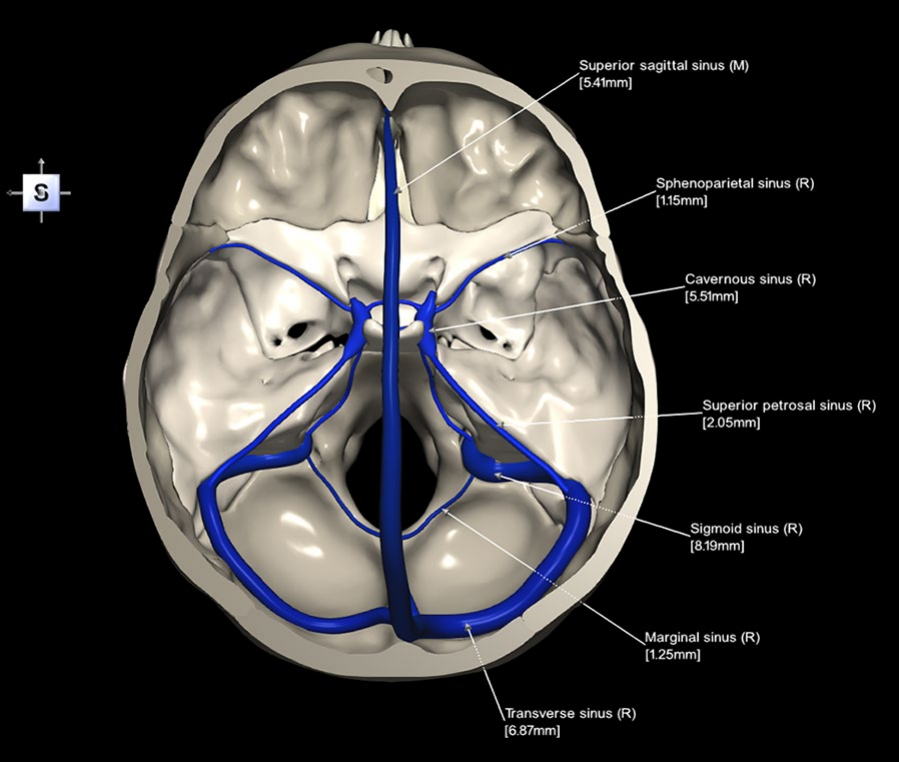
Skull base with the dural sinuses labeled (note that the vessels are also labeled with their diameters)

**Fig. 6:**
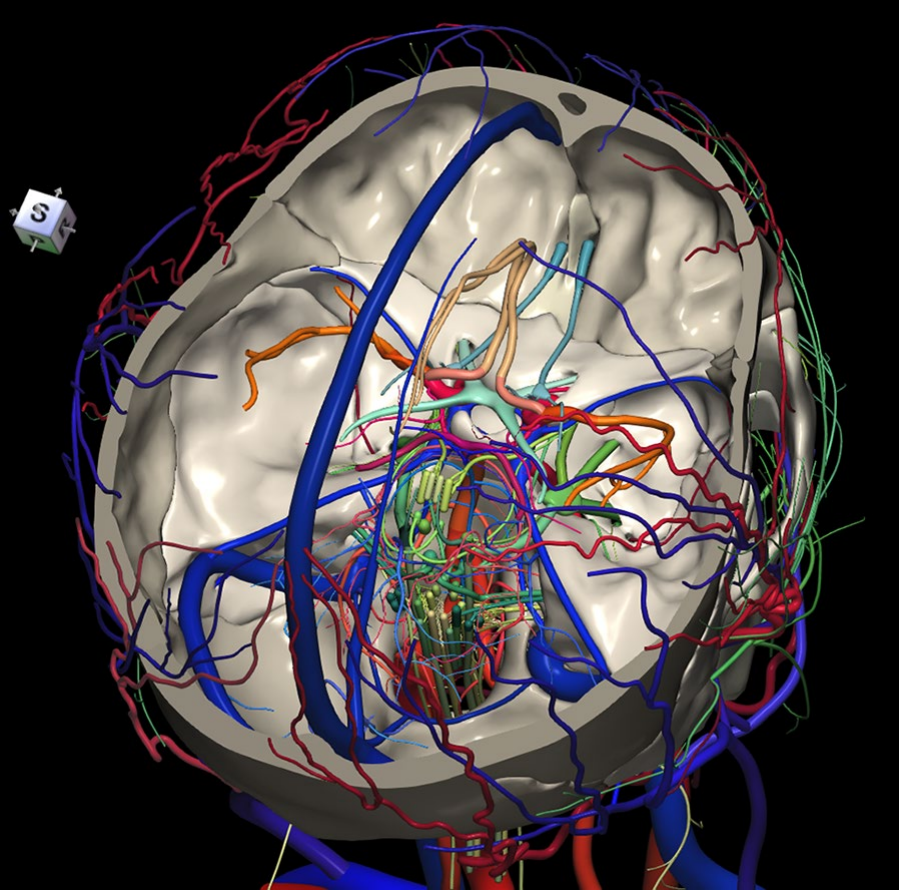
Skull base with the cranial nerves, dural sinuses, extracranial vessels and selected intracranial arteries and veins

**Fig. 7:**
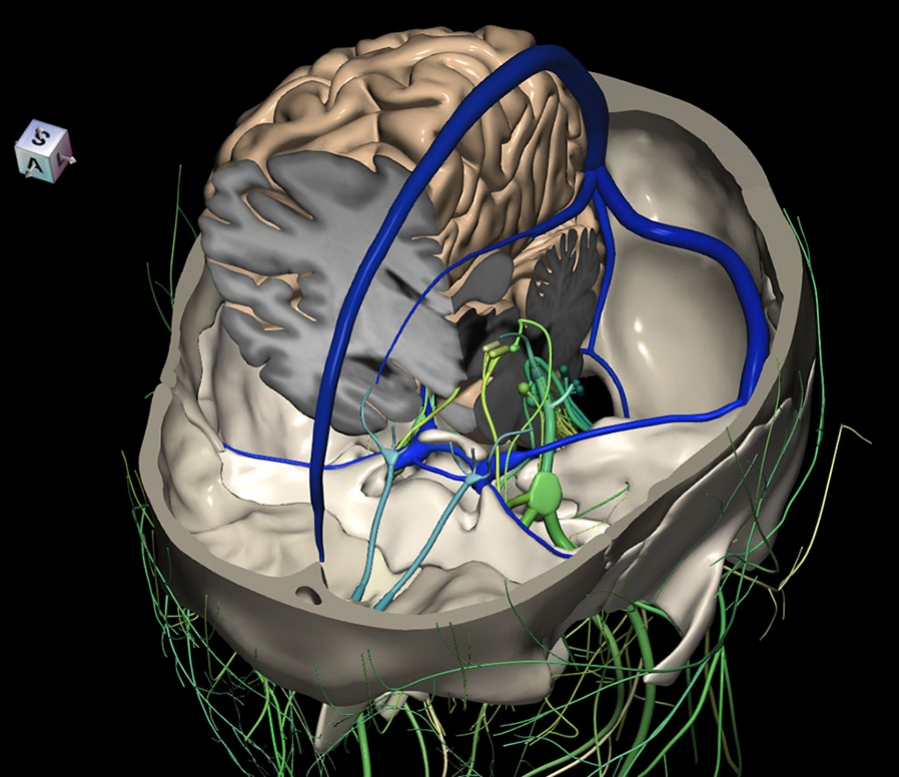
Skull base with an anteriorly dissected right hemisphere, cranial nerves with nuclei and dural sinuses

**Fig. 8:**
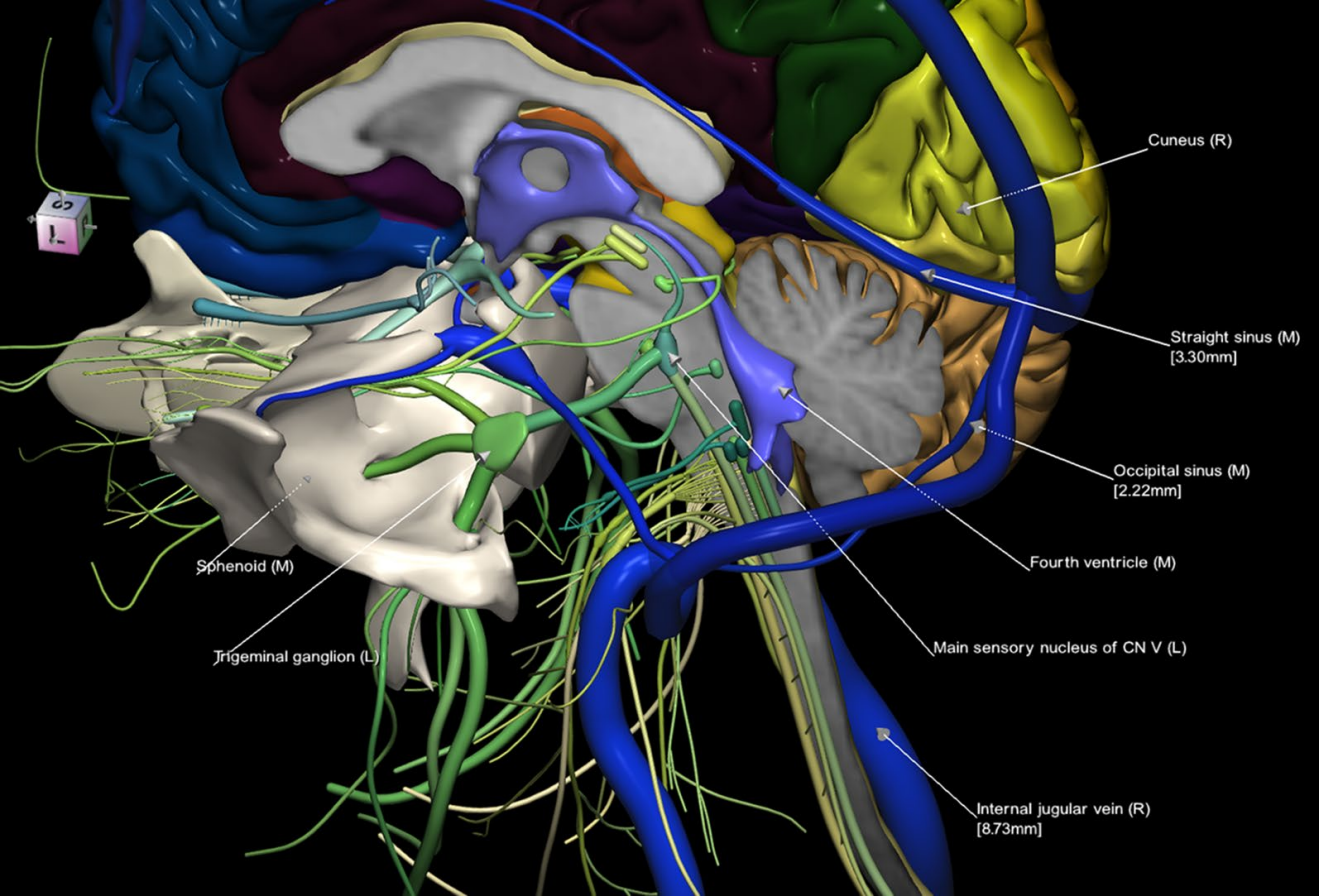
Sphenoid along with the brainstem and surrounding structures; some of them are labeled

**Fig. 9:**
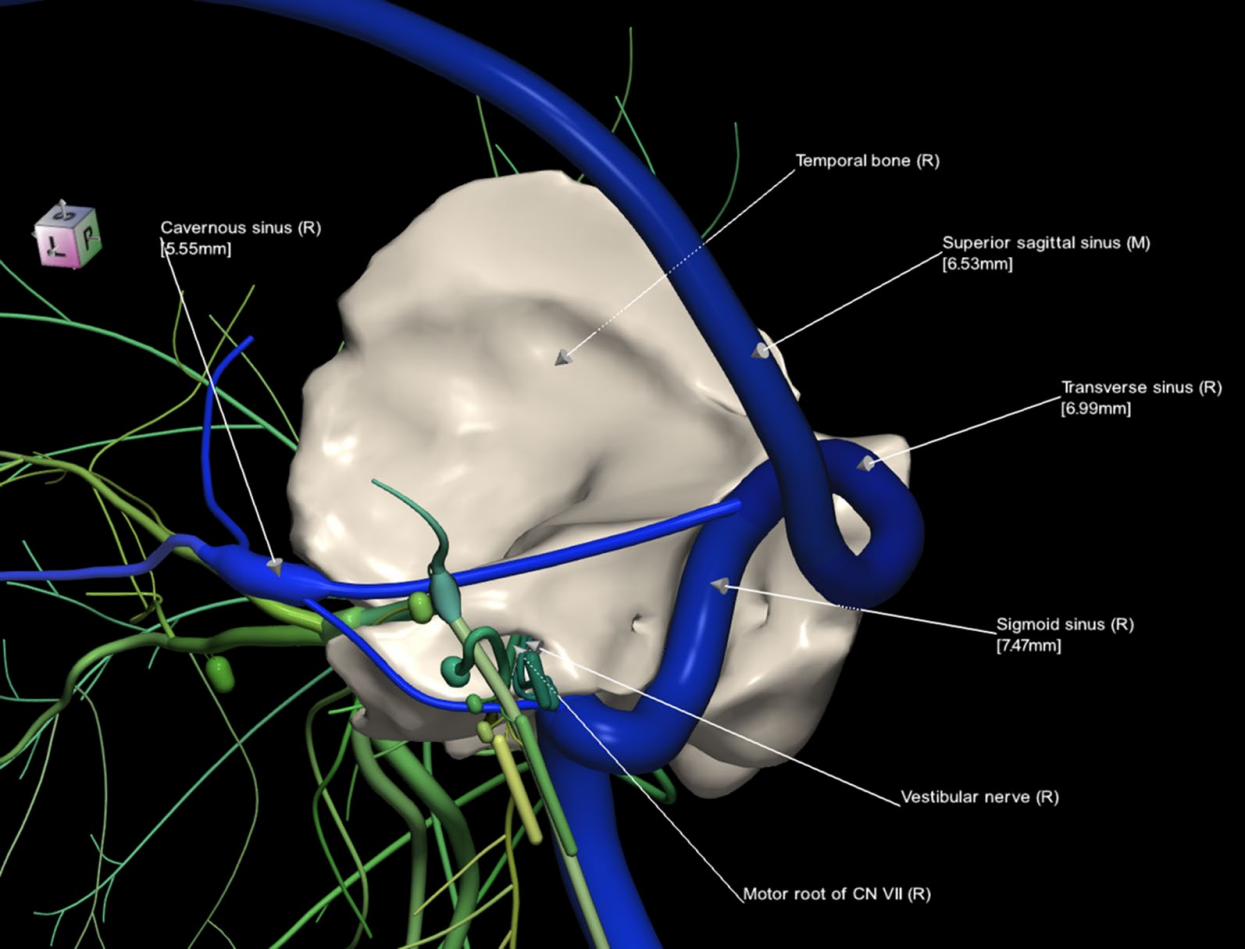
Temporal bone, cranial nerves and dural sinuses labeled

The 3D, electronic and stereotactic atlas of the adult human skull base has been designed and constructed and is illustrated in Figs. 1, 2, 3, 4, 5, 6, 7, 8, and 9. The content of the atlas is listed in Appendix. Figure 1 shows the internal (superior view) and external (inferior view) surfaces of the skull base. Figure 2 presents the internal surface of the skull base labeled with the anterior, middle, and posterior fossa, and with the color-coded bones. The skull base along with foramina, canals, and the cranial nerves is illustrated in Fig. 3. Figure 4 shows the skull base along with selected intra- and extracranial arteries. Figure 5 presents the skull base with the dural sinuses labeled. The skull base with the cranial nerves, dural sinuses, extracranial vessels, and selected intracranial arteries and veins is illustrated in Fig. 6. Figure 7 shows the skull base with an anteriorly dissected right hemisphere, cranial nerves with nuclei and dural sinuses. Individual bones along with their surroundings partially labeled are illustrated in Figs. 8 (the sphenoid) and 9 (the temporal bone).

This herein skull base atlas is a part of *The Human Brain, Head and Neck in 2953 Pieces* atlas [26], available publically at http://www.thieme.com/nowinski/ and www.WieslawNowinski.com/FreeBrainAtlas.

## Discussion

This work is discussed in terms of the background in skull base presentation and modeling, skull base atlas advantages and limitations, and future directions.

### Background

The existing skull base sources, including atlases, can be subdivided into four groups: textbooks, electronic media, 3D printed models and truly 3D electronic models. This work belongs to the last category. The skull base region has been extensively described in many sources, including textbooks of neuroanatomy [4], neuroradiology [2] and neurosurgery [3] as well as editions of Gray’s anatomy [5–7]. For description and illustration of spatial relationships in the skull base region, these sources use photographs, artistic drawings, and CT and MRI scans. There are several print atlases dedicated to the skull base region that mainly present predefined surgical approaches [1, 8, 9]. *Photo Atlas of Skull Base Dissection* by Wanibuchi et al. [9] is a richly illustrative textbook that contains high-quality photographs of cadaveric dissections with comprehensive anatomical views. It shows the anatomy as encountered through different surgical exposures according to the anterior, anterolateral, lateral, posterolateral, and posterior skull base approaches. *Skull Base and Related Structures: Atlas of Clinical Anatomy* by Lang [8] is a textbook addressing the development of skull base, cerebral arterial anatomy, skull base anatomy, and surgical approaches. The material has been illustrated with pictures of cadaveric specimens and drawings. The images are unlabeled or only partly labeled. *Atlas of Skull Base Surgery and Neurotology* by Jackler [1] focuses on surgical illustrations with text limited to describe operative techniques. Generally, the textbook materials including print atlases are static with no means of content handling electronically. Moreover, they are limited in content, presentation, view, labeling, and quantification of the skull base region.

These limitations are partly overcome by electronic media. The skull base region or some part of it is available for viewing and manipulation in some electronic atlases of the head and neck. *VOXEL*-*MAN* [12] comprises QuickTime movies of the brain and skull. As this is not a truly 3D atlas and the movies are reconstructed from volumetric data, consequently, anatomical structures cannot be freely selected or deselected for display and exploration, they are less detailed, many foramina are missing, and the atlas is not stereotactic. *A.D.A.M. Animated Dissection of Anatomy for Medicine* [10] consists of images created by medical illustrators. Its content is dissectible layer-by-layer. This is not a truly 3D atlas and it is not stereotactic. Primal’s *Interactive Head and Neck* [11] contains images and animations extracted from scans. The content can be viewed as user predefined cuttings of the skull base region only, without providing freely assembly and disassembly operations. Moreover, the head and neck vasculature is missing and the atlas is not stereotactic. SPL’s *Head and Neck Atlas* [13] contains a limited 3D skull model of the labeled anatomical structures generated from a single CT scan along with several predefined scene views as anatomy teaching files. The skull has a limited parcellation without small bones, cannot be dissected to expose the skull base, and there is not correlation of the 3D models with the sectional views. Cranial nerves, intracranial arteries and veins, foramina and canals, and important venous sinuses are missing. *Visible Body* [14] comprises a 3D skull model with parcellated bones that can be added or removed individually. Bony landmarks, canals, and foramina are segmented, colored, and fully labeled, which feature is missing in our atlas (i.e., manual labeling is still available (see, e.g., Fig. 2a); however, this is technically difficult (though feasible) when using truly 3D models to automatically recognize non-3D closed polygonal objects as empty spaces (canals or foramina) or point landmarks). However, *Visible Body* models were created by medical visualization professionals and were not derived from multiple tomographic acquisitions with various contents. Generally, in the abovementioned atlases, functions for measuring distances between structures, reading vessel diameter, and providing stereotactic coordinates are not available.

Obviously, a direct manipulation and exploration of a full, truly 3D skull base model is advantageous over 2D images, animations, and teaching files. A 3D model can be solid or electronic. In [15], a 3D printed model of the skull based has been employed for observation under the operative microscope. Another approach is to use a dedicated virtual reality environment to display, manipulate, and explore any data, in particular the skull base data [15, 16]. In [16], the Dextroscope, a virtual reality system (developed earlier in our laboratory [27]) is employed to display stereoscopically skull base cadaveric data and manipulate them naturally with both hands. The Dextroscope handles volumetric data which limits data spatial resolution. The use of virtual reality systems with electronic 3D skull base models is effective for surgical training to study, design, and execute various neurosurgical approaches.

### Advantages

To our best knowledge, this is the first truly 3D atlas of the adult human skull base that includes a fully parcellated and automatically labelable brain, skull, cranial nerves, and intra- and extracranial vasculature. The use of the same specimen allowed us to build from multiple scans the 3D anatomical models that are spatially consistent, and this will enable extending them consistently in the future with new scan acquisitions. The models are polygonal (surface-based) with a higher spatial resolution in comparison with volumetric models. These component models were earlier validated, so the created skull base model can be considered as typical (normal).

This 3D atlas allows the investigator to display the entire skull base itself (Figs. 1, 2) as well as any regions and structures inside and/or outside it (Figs. 3, 4, 5, 6, 7, 8, 9). Navigation is continuous with a real-time display. Within the skull base, any configuration of bone, brain, intra- and extracranial vessels, and cranial nerves can be composed (see examples in Figs. 4, 6, 8, 9). In particular, the brainstem region can be included and the access to it demonstrated (see Fig. 8). Navigation is supplemented with axial, coronal, and sagittal 2D images (displayed as 3D objects) of the MRI (MP-RAGE) scan along with brain cutting in 7 directions (see Fig. 7). The 3D anatomical scene or configuration assembled by the investigator can be viewed from any position and at a wide range of magnification. The composed skull base configuration can freely be labeled in terms of the selected components and label placement as well as any distances measured and vessel diameters read (see Fig. 5). All 3D closed objects are labeled automatically, including structures, bones, vessels and cranial nerves (see Figs. 5, 8, 9). Non-3D closed objects, such as canals, foramina, sutures, fossae, bony landmarks, and bony sinuses created as holes in the automatically labeled objects, can be labeled manually (see Fig. 2a). Moreover, stereotactic coordinates of any location or landmarks are provided, such as foramina and notches. These features facilitate to study and explore the skull base region. To our best knowledge, this is the first stereotactic atlas of the skull base region.

### Limitations

At present, there are no predefined surgical approaches in the atlas nor dedicated surgical tools, although the available manipulation tools allow the investigator to assemble and disassemble any region, and cut it in four planes (and 7 directions) in axial, coronal, sagittal, and viewing direction planes. There is no embedded tool to capture videos; the atlas only allows the user to save the composed image to an external file. The atlas does not contain any variants of the skull base region. There are no cross-sections for the CT scans, because of a difficulty in a very accurate spatial registration of the CT scan with multiple MRI scans due to geometric distortions and various artifacts (only 3D models were spatially registered followed by a subsequent editing in order to mutually fit all the components). Moreover, there are no diameters for foramina and cranial nerves.

*Future directions* will focus on extending the current anatomical model, adding skull base variants, and including predefined surgical approaches. Moreover, we plan to develop a low cost, virtual reality surgical simulator with stereoscopic view and natural, hand-controlled content navigation for laparoscopic skull base surgeries.

In summary, the created 3D interactive atlas of the adult human skull base is an easy and useful aid in studying, understanding, and teaching spatial relationships of the skull base anatomy. It allows the investigator to create any configuration, and explore and measure it, which enhances the understanding of the skull base region (including the cavernous sinus) just with a few mouse clicks, making it useful for medical students, residents, teachers, and clinicians. The atlas is a helpful tool to generate teaching materials, and a potential component of any skull base virtual reality surgical simulator.

## Appendix: atlas content

1. Brain parcellated into the cerebrum, cerebellum and brainstem along with white matter tracts; the cerebral cortex is parcellated into lobes, gyri, and sulci; the cervical spinal cord is also included.
2. Cranial nerves CN I to XII with cranial nerve nuclei.
3. Visual system.
4. Auditory system.
5. Intracranial arteries.
6. Intracranial veins.
7. Venous sinuses.
  - Superior sagittal sinus.
  - Inferior sagittal sinus.
  - Straight sinus.
  - Occipital sinus.
  - Cavernous sinus (anterior and posterior intercavernous sinuses).
  - Superior petrosal sinus.
  - Inferior petrosal sinus.
  - Transverse sinus.
  - Sigmoid sinus.
  - Marginal sinus.
  - Sphenoparietal sinus.
  - Confluence of sinuses.
8. Bones.
  - Ethmoid bone.
  - Sphenoid bone.
  - Occipital bone.
  - Frontal bone.
  - Temporal bone.
  - Auditory ossicles (malleus, incus, stapes).
9. Paranasal sinuses.
  - Maxillary sinus.
  - Ethmoid sinus.
  - Sphenoid sinus.
  - Frontal sinus.
10. Foramina.
  - Supraorbital foramen.
  - Infraorbital foramen.
  - Anterior ethmoidal foramen.
  - Posterior ethmoidal foramen.
  - Superior orbital fissure (incomplete foramen).
  - Inferior orbital fissure (incomplete foramen).
  - Foramen rotundum.
  - Incisive foramen.
  - Greater palatine foramen.
  - Lesser palatine foramen.
  - Foramen ovale.
  - Foramen spinosum.
  - Foramen lacerum.
  - Internal acoustic meatus.
  - Jugular foramen.
  - Foramen magnum.
  - Stylomastoid foramen.
  - Mastoid foramen.
  - Greater petrosal foramen.
11. Sutures.
  - Norma lateralis.
    (i) Coronal suture.
    (ii) Lambdoid suture.
    (iii) Occipitomastoid suture.
    (iv) Squamosal suture (parietomastoid suture).
    (v) Sphenofrontal suture.
    (vi) Sphenoparietal suture.
    (vii) Sphenosquamosal suture.
    (viii) Sphenozygomatic suture.
    (ix) Zygomaticotemporal suture.
    (x) Zygomaticofrontal suture.
  - Norma basalis.
    (i) Frontoethmoidal suture.
    (ii) Sphenoethmoidal suture.
    (iii) Sphenopetrosal suture.
  - Norma frontalis/ventricularis.
    (i) Sagittal suture (this suture is not part of the skull base atlas, but it is available in the skull atlas).
12. Canals.
  - Infraorbital canal.
  - Pterygopalatine canal.
  - Incisive canals.
  - Mandibular canal.
  - Pterygoid canal (vidian canal).
  - Semicircular canals (of the ear).
  - Optic canal.
  - Hypoglossal canal.
